# Le naevus bleu cellulaire atypique du poignet: à propos d'un cas et revue de la literature

**DOI:** 10.11604/pamj.2014.18.248.4291

**Published:** 2014-07-26

**Authors:** Hassan Boussakri, Jean Luc Roux, Luc Durand, Abdelhalim Elibrahimi, Abdelmajid Elmrini

**Affiliations:** 1 Institut Montpelliérain de la Main, Clinique Climentville, Montpellier, France; 2 Centre de Pathologie, Parc Euromédecine, Montpellier, France; 3 Service de Chirurgie Ostéo-Articulaire (B4), CHU Hassan II, Fès, Maroc

**Keywords:** Naevus bleu atypique, poignet, diagnostiques différentiels, Atypical blue nevus, wrist, differential diagnosis

## Abstract

Le naevus bleu cellulaire atypique est une entité pathologique rare et sa localisation au niveau du poignet est exceptionnelle. Il est Considéré comme une Variante à des caractéristiques intermédiaires entre le naevus bleu cellulaire typique et le naevus bleu malin, dont l’évolution est incertaine. Le but de notre travail est d'attirer l'attention sur cette variété lésionnelle rare et de discuter les diagnostiques différentiels, ainsi que décrire les aspects histologique et les options thérapeutiques possibles.

## Introduction

Le naevus bleu cellulaire atypiques est une nouvelle entité récemment décrite dans la littérature [1]. Considéré comme une Variante à des caractéristiques intermédiaires entre le naevus bleu cellulaire typiques et le naevus bleu malin, dont d’évolution est incertaine. IL se manifeste habituellement par la présence des cellules dépourvue de mitoses atypiques et de nécrose [2]. Le naevus bleu cellulaire peut subir exceptionnellement une transformation maligne pour devenir un naevus bleu malin caractérisé par des métastases ganglionnaires qui sont présentent dans 80% à 90% des cas [3, 4]. Nous rapportons une forme particulière et exceptionnelle d'une tumeur cutanée à travers la description d'une observation d'un garçon de 18 ans, présentant une masse unique asymptomatique sur son poignet droit. Une biopsie exérèse de la tumeur montrait un nævus bleu cellulaire atypique. Il n′y a eu aucun signe de récidive après 6 mois de recul après la chirurgie. Le but de notre travail est d'attirer l'attention sur cette variété lésionnelle rare et de discuter les diagnostiques différentiels. Ainsi que décrire les aspects histologiques.

## Patient et observation

Il s'agissait d'un patient âgé de 18 ans, droitier de latéralité, étudiant, ayant comme antécédents une pyélonéphrite pendant l'enfance traitée et guérie sans séquelle, sans autres antécédents pathologiques particuliers notamment pas d'antécédents familiaux de mélanome,admis en consultation spécialisée de la chirurgie de la main et du membre supérieure pour deux problèmes: une sensation d'instabilité de l’épaule droit et une formation saillante au niveau de la face postérieure du poignet droit. L'examen clinique a objectivé une épaule de morphologie et de trophicité musculaire normales et un test d'appréhension positive pour laquelle on a décidé une abstention thérapeutique avec une rééducation. Par ailleurs, l'examen du poignet a trouvé une tumeur saillante, palpable, mesurant de 5x3 cm de diamètre, d'aspect vasculaire, localisée niveau de la face postérieure du poignet droit en regard de l'articulation radio-carpienne. La lésion était connue depuis l'enfance et considérée comme un angiome sans remaniement cutané ni augmentation de volume, ni gène fonctionnel avec conservation de l’état général. La radiographie du poignet droit était sans particularité. Une biopsie exérèse chirurgicale mettait en évidence une tumeur rouge, bien limitée en profondeur et en périphérie.

**Figure 1:**
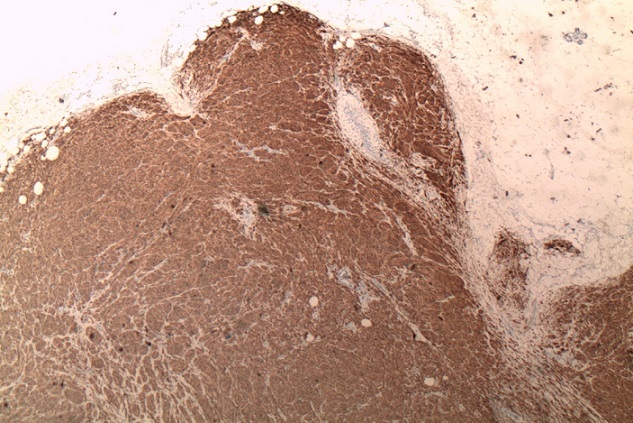
Faible grossissement (x25) de l'immonomarquage au mélana qui souligne la prolifération mélénocytaire en battant de cloche dans l'hypoderme

**Figure 2:**
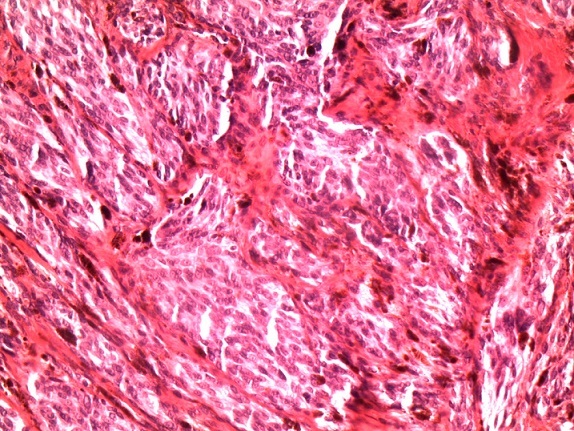
Fort grossissement (x200) à la coloration hémateine éosine qui montre que la prolifération mélanocytaire est constituée de cellules fusiformes agencées en faisceaux associés à du pigment mélanique avec atypies cytonucléaires minime et nucléoles de petite taille

L'examen anatomopathologique sur la pièce opératoire a objectivé une tumeur siégeant au niveau dermo-hypodermique, d'architecture lobulée, bien délimitée en périphérie (Figure 1). La prolifération était constituée de cellules ovalaires denses, pigmentées modérément atypiques. Les noyaux au contour irrégulier possédaient une chromatine fine et un petit nucléole. Dans le derme réticulaire, des cellules dendritiques fusiformes ou étoilées étaient chargées de mélanine (Figure 2). L'activité mitosique ne dépasse pas deux par plan de coupe. Il n'ya pas de zones de nécrose visible ou de phénomène inflammatoire surajouté.

L’étude immuno-histochimique réalisée montre un marquage des cellules tumorales positives avec l'anti-corps HMB45. Le taux de prolifération cellulaire estimé sur le Ki 67 ne dépasse pas 5% de noyaux marqués avec des rares mitoses sur PPH3. A noté qu'il n′y a eu aucun signe de récidive à 6 mois de recul après la chirurgie. Le diagnostic du naevus bleu cellulaire atypique a été faite sur la base des caractéristiques cliniques et surtout les résultats anatomopathologiques de biopsie de la peau qui a permet de confirmé le diagnostic.

## Discussion

Le naevus bleu cellulaire atypique est une entité histologique qui n'est pas encore bien définie, mais la majorité des publications [3, 5, 6] décrivent cette pathologie comme une entité avec des caractéristiques histologiques différentes du naevus bleu malin dont la malignité est sure et du naevus bleu cellulaire dont l’évolution est incertaine. Il se définit par la présence d'atypies cytonucléaires sans mitoses atypiques associées. Le diagnostique différentiel à l’étape histologique du naevus bleu cellulaire atypique est le naevus bleu malin développé sur un naevus bleu cellulaire est parfois difficile.

Les éléments épidémiologiques sont importants à prendre en considération dans la prise en charge thérapeutique mais surtout dans le diagnostic positif. Typiquement le naevus bleu cellulaire atypique touche une jeune femme avec sexe ratio du 9/6, et le diagnostic clinique fait généralement a partir de l’âge de 20 ans [5, 7]. Ce qui rend notre observation importante d'une part est que le diagnostique fait a l’âge jeune (18 ans), aussi le sexe masculin, mais surtout localisation au niveau de la main. A limite de notre recherche bibliographique aucun cas similaire de naevus bleu cellulaire atypique avec une localisation de la main n'a pas été décrits dans la littérature, par ailleurs Les localisations des cas décrite dans la littérature de naevus bleu cellulaire atypique sont par ordre de fréquence: la région sacrée, le cuir chevelu et en fin les membres [6]. Concernant La taille tumorale du naevus bleu cellulaire atypique, elle varie de 1 à 4 cm selon les auteurs sans différence avec la taille du naevus bleu malin [4, 8], mais Au delà de ces dimensions la malignité est suspectée.

L'examen anatomopathologique sur la pièce opératoire est le seul moyen qui permet de confirmer le diagnostic exacte de naevus bleu cellulaire atypique. Tran et al [6], ont rapporté sur un examen microscopique sur une série de neuf cas, d'ailleurs c'est la plus grande série de la littérature, la présence de mitoses peu nombreuses (1 à 2 mitoses / 10 champs), et qui ne sont jamais atypiques, La nécrose peut exister mais très limitée, de même les atypies nucléaires avec proéminence des nucléoles, et l'infiltrat inflammatoire modéré. La délimitation périphérique avec un aspect de « pushing margins » n'est pas un critère de malignité. Les critères majeurs actuellement reconnus du naevus bleu malin sont la nécrose tumorale en foyer ou étendue [9] et les mitoses atypiques [5, 7, 10]. Le nombre de mitoses normales ou atypiques peut être faible à élevé, soit de moins de 1 mitose / 10 champs, soit jusqu’à 40 mitoses/ 10 champs [7, 10]. La présence de mitoses atypiques est le signe pathognomonique de malignité [5, 7, 10, 11], mais inconstamment retrouvée même dans les cas de naevus bleu malin avec métastase [7, 12]. Dans cette lésion maligne, les atypies cytologiques sont d'intensité variable, discrètes à marquées jusqu'au pléomorphisme nucléaire. Les noyaux peuvent être hyper-chromatiques et comporter un ou plusieurs nucléoles volumineux. L'augmentation rapide du volume tumoral [10] ou des modifications morphologiques récentes comme l'ulcération cutanée [9] font suspecter la transformation maligne. Dans notre cas, la tumeur étant dépourvue de mitoses atypiques, de nécrose, d'atypies majeures, d'invasion tumorale et de métastase au moment du diagnostic, nous avons exclu le diagnostic de malignité.

Le traitement du naevus bleu cellulaire atypique est l'exérèse chirurgicale totale avec des limites d'exérèse saines. Celui du naevus bleu malin nécessite des marges chirurgicales effectuées suffisamment à distance, comme pour le mélanome. La chimiothérapie n'a pas fait la preuve de son efficacité pour le naevus bleu malin.

## Conclusion

En conclusion, le naevus bleu cellulaire atypique est une forme histologique particulière, et la localisation au niveau du poignet est rare. Il est important de reconnaître cette entité frontière, car son évolution et son pronostic sont difficiles à déterminer. Nous recommandons toujours une exérèse chirurgicale et une surveillance clinique à long terme.

## References

[1] Ryou JH, Lim TW, Lee MH, Haw CR (2000). Pedunculated atypical cellular blue nevus. J Dermatol..

[2] Ahhot P, Rivet J, Saliou C, Bailly C, Brunevalb P (2000). Le naevus bleu cellulaire atypique, à propos d'un cas. Ann Pathol..

[3] Goetteg DK, Robinson JW (1980). Atypical cellular blue nevus. J As-soc Milit Dermatol..

[4] Boi S, Barbareschi M, Cristofolini M (1989). Malignant cellular blue nevus with true nodal metastases. Pathologica..

[5] Gonzales Campora R, Galera Davidson H, Vasquez Ramirez FJ, Diaz cano S (1994). Blue nevus: classical types and new related entities, diagnostic review. Path Res Pract..

[6] Tran TA, Carlson JA, Basaca PC, Mihm MC (1998). Cellular blue nevus with atypia: a clinicopathologic study of nine cases. J Cutan Pathol..

[7] Connelly J, Smith JL (1991). Malignant blue nevus. Cancer..

[8] English JC, MC Cullough ML, Grabski WJ (1996). A pigmented scalp nodule: malignant blue nevus. Cutis.

[9] Templecamp RE, Saxe N, King H (1988). Benign and malignant cellular blue nevus: A clinicopathological study of 30 cases. Am J Dermatopathol..

[10] Ozgur F, Akyurek M, Kayikcioglu A, Barista I, Gokoz A (1997). Metastatic malignant blue nevus, a case report. Ann Plastic Surg..

[11] Aloi F, Pich A, Pippione M (1996). Malignant cellular blue nevus: a clinicopathologic study of 6 cases. Dermatology..

[12] Spatz A, Zimmermann U, Bachollet B, Pautier P, Michel G, Duvillard P (1998). Malignant blue nevus of the vulva with late ovarian metastasis. Am J Dermatol..

